# Modeling Crop Genetic Resources Phenotyping Information Systems

**DOI:** 10.3389/fpls.2019.00728

**Published:** 2019-06-21

**Authors:** Christoph U. Germeier, Stefan Unger

**Affiliations:** ^1^ Institute for Breeding Research on Agricultural Crops, Julius Kühn Institute, Federal Research Centre for Cultivated Plants, Quedlinburg, Germany; ^2^ Data Processing Department, Julius Kühn Institute, Federal Research Centre for Cultivated Plants, Quedlinburg, Germany

**Keywords:** plant genetic resources, phenotyping, documentation, entity relationship models, class models, work flows, web applications

## Abstract

Documentation of phenotype information is a priority need in biodiversity, crop modeling, breeding, ecology, and evolution research, for association studies, gene discovery, retrospective statistical analysis and data mining, QTL re-mapping, choosing cultivars, and planning crosses. Lack of access to phenotype information is still seen as a limiting factor for the use of plant genetic resources. Phenotype data are complex. Information on the context, under which they were collected, is indispensable, and the domain is continuously evolving. This study describes comprehensive data and object models supporting web interfaces for multi-site field phenotyping and data acquisition, which have been developed for Central Crop Databases within the European Cooperative Programme for Plant Genetic Resources over the years and which can be used as blueprints for phenotyping information systems. We start from the hypothesis, that entity relationship and object models useful for software development can picture domain expertise, similar as domain ontologies, and encourage a discussion of scientific information systems on modeling level. Starting from information requirements for statistical analysis, meta-analysis, and knowledge discovery, models are discussed in consideration of several standardization and modeling approaches including crop ontologies. Following an object-oriented modeling approach, we keep data and object models close together and to domain concepts. This will make database and software design better understandable and usable for domain experts and support a modular use of software artifacts to be shared across various domains of expertise. Classes and entities represent domain concepts with attributes naturally assigned to them. Field experiments with randomized plots, as typically used in the evaluation of plant genetic resources and in plant breeding, are in the focus. Phenotype observations, which can be listed as raw or aggregated data, are linked to explanatory metadata describing experimental treatments and agronomic interventions, observed traits and observation methodology, field plan and plot design, and the experiment site as a geographical entity. Based on clearly defined types, potential links to information systems in other domains (e.g., geographic information systems) can be better identified. Work flows are shown as web applications for the generation of field plans, field books, templates, upload of spreadsheet data, and images.

## Introduction

Information systems become increasingly important tools in biological sciences and cover a considerable part in a recent review on next-generation phenotyping (Cobb et al., 2013). The biodiversity (Wieczorek et al., 2012; Hardisty et al., 2013) and the crop modeling communities (Bostick et al., 2004; White et al., 2013) have been identified as strong and early proponents for biological information systems (Tinker and Yan, 2006). Phenotyping and genotyping are of main interest in crop science and breeding, plant traits also in ecology and evolution research (Kattge et al., 2011). Tinker and Yan (2006) address needs for efficient storage and retrieval of crop performance data to increase their value for exploration, reporting, crop modeling, planning crosses, and retrospective statistical analysis. They envision automatic generation of orthogonal subsets for statistical analysis based on information on experimental context, support for continuous QTL re-mapping, association studies, gene discovery, and data mining in crop science and plant breeding. Early initiatives started with stand-alone information systems in breeding programs (e.g., Haley et al., 1999; Lang et al., 2001; Heckenberger et al., 2008), or web-based systems for decision support to choose cultivars (Jensen, 2001), or for genetic resources management by the USDA (USDA Agricultural Research Service, 2015a) and the CGIAR (McLaren et al., 2005). Recently, these have culminated into the GRIN-Global project, covering multiple types of genebank data (USDA Agricultural Research Service, 2015b).

Nevertheless, the Second Report on the State of the World’s Plant Genetic Resources for Food and Agriculture (PGRFA) still highlighted lack of access to information, especially characterization and evaluation (phenotyping) data as most important limiting factor for an increased use of PGRFA in agriculture, horticulture, crop improvement, and research (FAO, 2010). Tinker and Yan (2006) mention an exploratory and a reporting mode needed to discover an information system: The exploratory mode summarizes results related to a keyword (e.g., a cultivar name). The reporting mode generates lists responding to specific queries (e.g., for stress tolerance or special nutritional quality). Jensen (2001) points out the requirement for mechanisms to compare and rank genotypes according to multiple traits, typically involving attribute-centric queries (Dinu and Nadkarni, 2007). Haley et al. (1999) demand simultaneous assessments of multiple traits on a standardized scale and from sets of comparable data gathered in one environment, user-specified prioritization of traits, tabulation of specific deficiencies, and summaries across multiple environments.

Consortia for Agricultural Systems Applications (Bostick et al., 2004; White et al., 2013) and Agrotechnology Transfer (Jones et al., 2003) have been established to promote a better use of expensive site- and season-specific field experiments. Crop models integrate knowledge about soil, climate, crops, and management to better understand their function, allow transfer of results to other agro-ecological conditions, and predict crop behavior (Jones et al., 2003). Models for 42 crops have been integrated with modules for weather, soil (water, carbon and nitrogen contents, temperature), plant, atmosphere, management, pests, and diseases, in the DSSAT-CSM software (Hoogenboom et al., 2017). Bostick et al. (2004) presented a first version of a web application (ICASA Data Exchange) targeted to stimulate the reuse of various types of field experiment data for agricultural systems modeling in the International Consortium for Agricultural Systems Applications (ICASA). It provides metadata on data owners, experiment, environment, crop, management, measurement, file, and publication information for uploaded data files. Several papers have been published on data models for crop genetic resources phenotyping and breeding databases (Lee et al., 2005; Yan and Tinker, 2007; Heckenberger et al., 2008; Fabre et al., 2011; Jung et al., 2011). None of these systems has been widely used (Vankadavath et al., 2009; White et al., 2013; Weise et al., 2017) to provide a reference for phenotyping as, e.g., GenBank provides for genotyping. Obviously, they have drawbacks in comprehensiveness or usability, as the domain is complex. Lack of modularization often is an impediment for the reuse of software. Modules should work independently of each other in different contexts. GERMINATE, introduced as data model for genetic and phenotypic data from a generic marker viewpoint (Lee et al., 2005), has been reused to build the Hordeum and Triticeae Toolboxes (Blake et al., 2012) and recently revised to the new version of Germinate 3 (Shaw et al., 2017). A standardized and modularized RESTful web service application programming interface (Breeding API, https://wiki.brapi.org/index.php/BrAPI) is currently developed for all types of breeding relevant data (germplasm, genotyping, phenotyping).

Information system ontologies (Evermann and Wand, 2005) and respective tools have been employed to tag phenotyping data for search and computer algorithms (Cobb et al., 2013). Specific crop ontologies (Bioversity International, 2011) have been set up as an open project, built on other ontologies, e.g., Plant Ontology (Avraham et al., 2008; Planteome, 2016), Environment Ontology (Buttigieg et al., 2013), and others within the Open Biological and Biomedical Ontologies^1^. Currently, most of the ontologies available from the crop ontology service have a single authorship, and multiple trait ontologies exist for crops like barley and soybean, indicating that they are not yet a community resource. Ontologies have similarity to domain models or domain-specific languages (Ceh et al., 2011), but their use in software engineering is not yet fully understood and implemented by tools (Fogh et al., 2005; Cranefield and Pan, 2007). Köhl and Gremmels (2015) used Plant Ontology (for entities) and Plant Trait Ontology (partly for attributes) to compile a scheme database supporting a web application, which generates phenotyping schemes with controlled vocabularies. Hannemann et al. (2009) describe tools to integrate ontologies into growth chamber experiments design and documentation. Munir and Anjum (2018) review recent approaches and tools using ontologies with relational databases, primarily to improve information retrieval with ontology based user interfaces. They describe tools for ontology to database mapping, which link pre-existing databases and ontologies, and for database to ontology transformations, which generate an ontology from a database or vice versa (Vysniauskas and Nemuraite, 2006).

Here we describe data and object models, which have been developed since 2000 for Central Crop Databases within the European Cooperative Programme for Plant Genetic Resources (ECPGR), used for web-based query interfaces (Germeier and Frese, 2001, 2004) and more recently to build web interfaces for workflows coordinating multi-site characterization and evaluation (phenotyping) of PGRFA and respective data acquisition. We start from the hypothesis that entity relationship (ER) database models, class, and object models in scientific software can be appropriately designed as representations of the scientific domain and its real-world concepts. This will facilitate the comprehension of software concepts by domain experts and the modularization of scientific software infrastructures in accordance with different fields of domain expertise. It will further promote the reuse of functionality and of scientific data and a closer connection between general software development and simulation modeling (Papajorgji et al., 2004). Finally, it will be also an outcome of simple database to ontology or ontology to data model transformations (Vysniauskas and Nemuraite, 2006; Munir and Anjum, 2018). While working on our models, we had not yet any ontology – ER mapping tools (Munir and Anjum, 2018) at hand.

With this emphasis and based on requirements for statistical analysis of phenotyping data, we discuss models and work flows encountered in our characterization and evaluation projects (Morcia et al., 2013; Murariu et al., 2013; Redaelli et al., 2013, 2016; Tumino et al., 2016) in consideration of suggestions for standardization, other modeling approaches, and crop ontologies. We suggest them as blueprints, which can be used independent of concrete software implementations. The latter undergo short lifecycles determined by their developing technology platforms.

We strictly focus on a phenotyping information module covering field experiments with randomized plots, as typically used in the evaluation of plant genetic resources and in plant breeding. Fahlgren et al. (2015) mention advantages of field phenotyping platforms, as are growing crop-sized plants under natural settings. Araus and Cairns (2014) refer to the lack of quantitative trait loci and candidate genes detected in controlled environments to translate into gains in the field. Our modeling approaches (Figures 2–5) can be taken as a blueprint for the design of phenotyping information systems documenting field experiments in crop and plant sciences.

## From Standards To Models

The crop modeling community made first attempts to standardize phenotype data, e.g., in the International Consortium for Agricultural Systems Applications (ICASA; Bostick et al., 2004; White et al., 2013). A similar activity in current plant phenotyping networks defines Minimum Information About a Plant Phenotyping Experiment (MIAPPE) standards (Ćwiek-Kupczyńska et al., 2016), which are currently implemented with the Breeding API. For PGRFA, Endresen and Knüpffer (2012) included terms for field observations (measurement, measurement method, and experiment) into a genebank extension of the Darwin Core data exchange format for the Global Biodiversity Information Facility (GBIF).

While standardization counts on an agreement to choose a dedicated approach out of equivalent variants, modeling has the ambition to provide the best state of the art representation of the knowledge in a domain. Thus, requirements for modeling are higher (Swertz and Jansen, 2007). Standards can be rigid and finally unable to cope with scientific progress, while information systems need sufficient flexibility that “today’s data can contribute to tomorrow’s opportunities” (Fogh et al., 2005; Lee et al., 2005; Tinker and Yan, 2006). Models remain subject to scientific discussion. This causes change management in information systems but guarantees the flexibility required, the intelligibility for domain experts and reusability even for additional purposes, e.g., simulation modeling (Papajorgji et al., 2004).

**Figure 1:**
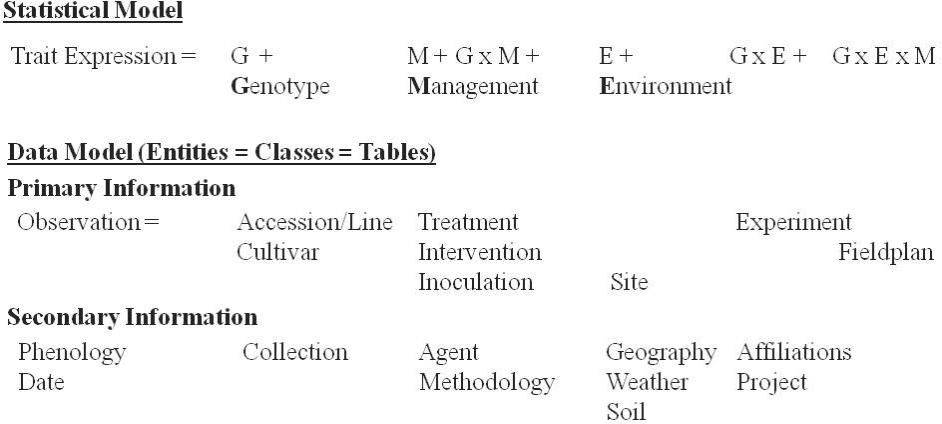
Levels of modeling in the phenotyping domain from requirements for statistical analysis to implementation of an information system as entities and classes.

Modeling comes into place on several levels of information management (Figure 1). On the highest level of abstraction are models for statistical analysis and knowledge discovery (Piepho et al., 1998; Malosetti et al., 2013). These determine what information is needed to create knowledge out of the data. At the bottom, static conceptual models (domain models) are used for database and software design. These are represented by entity-relationship and class diagrams, respectively (Evermann and Wand, 2005). Crop models as simulation or predictive models depict a mechanistic understanding of crop behavior and allow its prediction from input parameters like weather, soil, genotypes, experiment conditions, and measurements (Jones et al., 2003). Papajorgji et al. (2004) stressed the interrelationships of software modeling and simulation modeling and recommended to use tools from the software engineering community like Unified Modeling Language (UML) and component-based approaches also for simulation modeling.

Statistical or predictive phenotype models contain genotype, environment, and management as main factors and respective interactions (Kropff and Struik, 2002; Hatfield and Walthall, 2015). These factors can be further analyzed by the various classes of primary and secondary information shown in Figure 1. Genotypes in the PGRFA domain are accessions in genebank collections, breeding lines and cultivars. Management factors are agronomic interventions (soil tillage, irrigation, fertilization, plant protection) and experimental treatments differing for the experimental factors according to the scope of the study. These can include artificial inoculation with diseases or symbiotic organisms, requiring documentation of agents (strains). Also, the observation methodology belongs to the management factor. The geographic location of the experiment site with weather and soil conditions (cf. Figure 4), and on a small geographic scale, the spatial design of the field plan creates the environment. Experiments are affiliated with research projects and institutions (Heckenberger et al., 2008; Ćwiek-Kupczyńska et al., 2016).

## Generalization or Specialization

Generalization (abstraction) and specialization (level of detail) are critical points for modeling. Generalization has been put forward as a target improving reuse of data. Lee et al. (2005) described five modules (Passport, General, Data Integration, Information, and Datasets) to generalize over phenotype and genotype data, getting them into the same data structure in a module Datasets. Names of modules and several tables, especially in module “Datasets” did not intuitively correspond to objects known in the phenotyping and genotyping domains but referred to technical features of the data (e.g., “data types,” “data,” “metadata”). Names not referring to domain concepts but technical features are indicative for over-generalization or over-normalization. These limit understanding and reuse of information components and the modularization along a specialization of expertise. The conceptual ambiguities lead to a variety of data storage implementations (Lee et al., 2005). We consider phenotyping and genotyping sufficiently different domains and communities that they deserve specific modeling and modules. Steinbach et al. (2013) treat collection data in a genetic resources module, phenotype data from genotype-environment interaction studies in a phenotype, and genome data in several separate modules (sequence, genetic map, polymorphism, genome, transcriptome). Also, the recent version of Germinate 3 has separate marker/genotype and phenotype/field trials schemata (Shaw et al., 2017).

**Figure 2:**
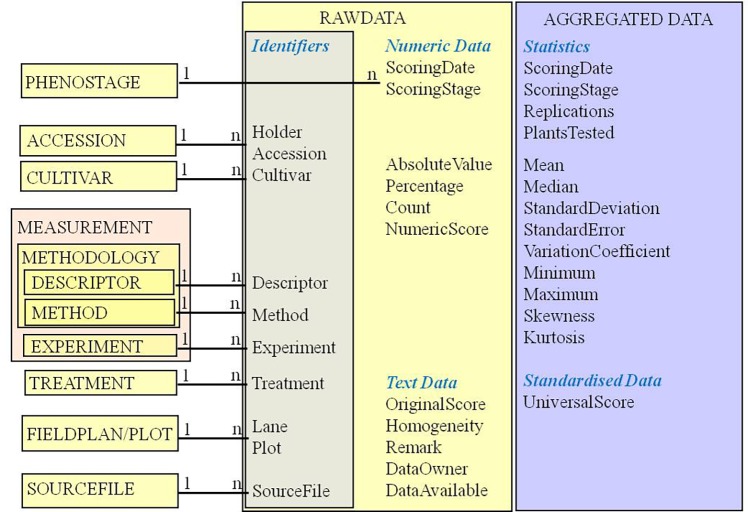
The phenotype observation as raw or aggregated data – its attributes of different types (identifiers and foreign keys, numeric data, strings, statistics and standardized harmonized or ranking data) and related descriptive metadata concepts. Entity names are written in capitals; attribute names within RAWDATA and AGGREGATED DATA in mixed case.

Kattge et al. (2011), from an ecologist point of view, stress the nesting of observations in a hierarchy. Higher levels form the context for lowers (e.g., stand – individual – leaf – cell). Including environmental context and even taxonomy into this nesting, they provide a generic model for observations on plant and environment with the elements entity, observation, measurement, characteristic, and measurement standard. It resembles the “entity-attribute-value” (EAV) or “vertical design,” another pattern discussed for generalization (Billiau et al., 2012; Köhl and Gremmels, 2015). Avoiding fix defined data structures by modeling object attributes as entries, e.g., of property association tables (Jung et al., 2011), it also appears as a simple solution for mapping controlled vocabulary (thesauri and ontologies) into information systems (Billiau et al., 2012). Yet, deviating from relational and object-oriented design principles, it is not well supported by database management and software tools; e.g., queries for multiple attributes require cumbersome auto-referencing approaches (Corwin et al., 2007). Dinu and Nadkarni (2007) mention clear indications, under which vertical design can be used: sparse and volatile data. The EAV structure needs support by a system of relational metadata and metadata-driven software, and it again should represent a well-understood domain concept (e.g., a phenotypic observation, as shown in Figure 2).

Here we follow an object-oriented design, keeping data and object models close together and to domain concepts. Classes and entities by their naming are easily grasped by domain experts and cover attributes naturally assigned to these concepts in the domain. This will also result from transformations between ER, object models and ontologies (Vysniauskas and Nemuraite, 2006; Munir and Anjum, 2018).

Natural identifiers (often available as compound keys) are preferred to surrogate keys. Only meaningful keys implement meaningful integrity rules. Modularization will give different communities (e.g., genebanks, phenotyping, genotyping, breeding, environmental science, and geography) freedom to manage their data within own namespace and ontology. Interoperability of information from different domains does not result from pressing their data into a highly abstracted data structure, but by proper interfaces (APIs) to link clearly defined types into various information systems driven by different domains (cf. Figure 4).

## A Data Model For Phenotyping (Characterization and Evaluation) Data

Krajewski et al. (2015) define phenotyping as any quantitative or qualitative measurement of traits at levels from single cells, plants, field plots, up to ecosystems, and a plant phenotyping experiment as a set of experimental units with assigned levels of factors, resulting in a treatment and block structure within an experiment design. They recommend distinguishing factors related to “biosources” (accessions in PGRFA documentation) from those of real treatments (agents modifying the environment). van Evert et al. (1999) describe a data model for agro-ecological research data, with a broad scope on objects, which can be subject to field experiments. Köhl et al. (2008) and Heckenberger et al. (2008) enter into details of the genesis of plant lines (breeding processes like propagation, crossing, transformation). Here we focus on accessions in terms of the Multicrop Passport Descriptors (Alercia et al., 2015) as biosources according to Krajewski et al. (2015) and targets for phenotyping: genebank accessions, breeding lines, populations, or cultivars. These materialize as seed stock (stock in Jung et al., 2011) in a working collection. Their genesis is seen in the scope of separate information modules on germplasm and breeding management. Samples from a single plant, a tissue, up to pooled accessions or populations (Lee et al., 2005) can be referenced in a methodology description if of interest outside a separate Laboratory Information and Management System (LIMS). Yan and Tinker (2007) show basics of phenotypic data as a “context oriented observation library”. We follow these but develop more complex structures, where considered necessary.

### The Phenotypic Observation – Core of the Data Structure

The phenotypic observation has been defined as association of a trait with an observed value at a defined time (Krajewski et al., 2015) or as measurements taken on an object at the same time (Kattge et al., 2011). Observations as raw or aggregated data (Heckenberger et al., 2008; Blake et al., 2012) refer to trait expressions in an experimental unit (e.g., a plot), observed by measurement or estimation. They are modeled in EAV-like data structures (Billiau et al., 2012; Köhl and Gremmels, 2015): lists of observed trait expressions (raw or aggregated) in various data formats (numbers, symbols, words) are linked into a relational system of explanatory metadata (Dinu and Nadkarni, 2007). These describe accessions or cultivars identified by reference to the holding institute and an accession number or name, measurements referring to an observation methodology with trait descriptors (from descriptor lists or trait ontologies), methods (analytical protocols, classification schemes), experiments, treatments, field plots, and archived data source files (Figure 2). Kattge et al. (2011) use the term measurement for observations as presented here. Their observation entity links measurements in multiple dimensions of one object (hierarchy from environment to cell) in a certain time.

Trait observations need reference to an observation date and a phenological stage of the plant during observation (Billiau et al., 2012; White et al., 2013). For statistical re-evaluation (Blake et al., 2012) and long-term comparison (Keilwagen et al., 2014), data should be available in original states and formats (raw data, original score). Trait expressions are documented in different data types: numbers for measurements and counts; strings for morphological descriptions. For calculation, aggregation, sorting, and validation purposes, it is advantageous to foresee different fields for different data types (Dinu and Nadkarni, 2007): measured (absolute) values, percentages, counts, numeric scores, and text scores. Examples can be found easily, where more than one data type applies to an observation (e.g., surviving or diseased plants as absolute number or percentage of a target population). A coding system for homogeneity or heterogeneity in a plot has been proposed (van Hintum, 1993). Lee et al. (2005) designed different tables for each data type, including arrays for storing marker data. We consider one observation table for a specific aggregation level, with separate fields for different phenotypic result types most intuitive and easy to use with external (e.g., statistic) software. Further attributes refer to the owner of the data and their availability for different user types.

For presentation purposes, data aggregated at least to means of field replications in an experiment are preferred. Basic descriptive statistics (mean, median, standard deviation or error, range, skewness, kurtosis) should be given along with the number of replications and/or plants tested. Blake et al. (2012) mention means and summary statistics (range, standard error of mean, probability value for *F* test, outlier detection). Harmonizing and simplifying transformations or ranks (universal score, e.g., Grades 1–9) can give an impression on the first glance but do not fulfill scientific documentation requirements, especially not over long periods of time, when reference values are floating. Cobb et al. (2013) recommend storing data in a raw state for use in analyses, even when more robust Bayesian approaches are used. Keilwagen et al. (2014) suggest a normalized rank product for the comparison of measured traits over long periods of time.

### Traits, the Targets for Observation

**Figure 3:**
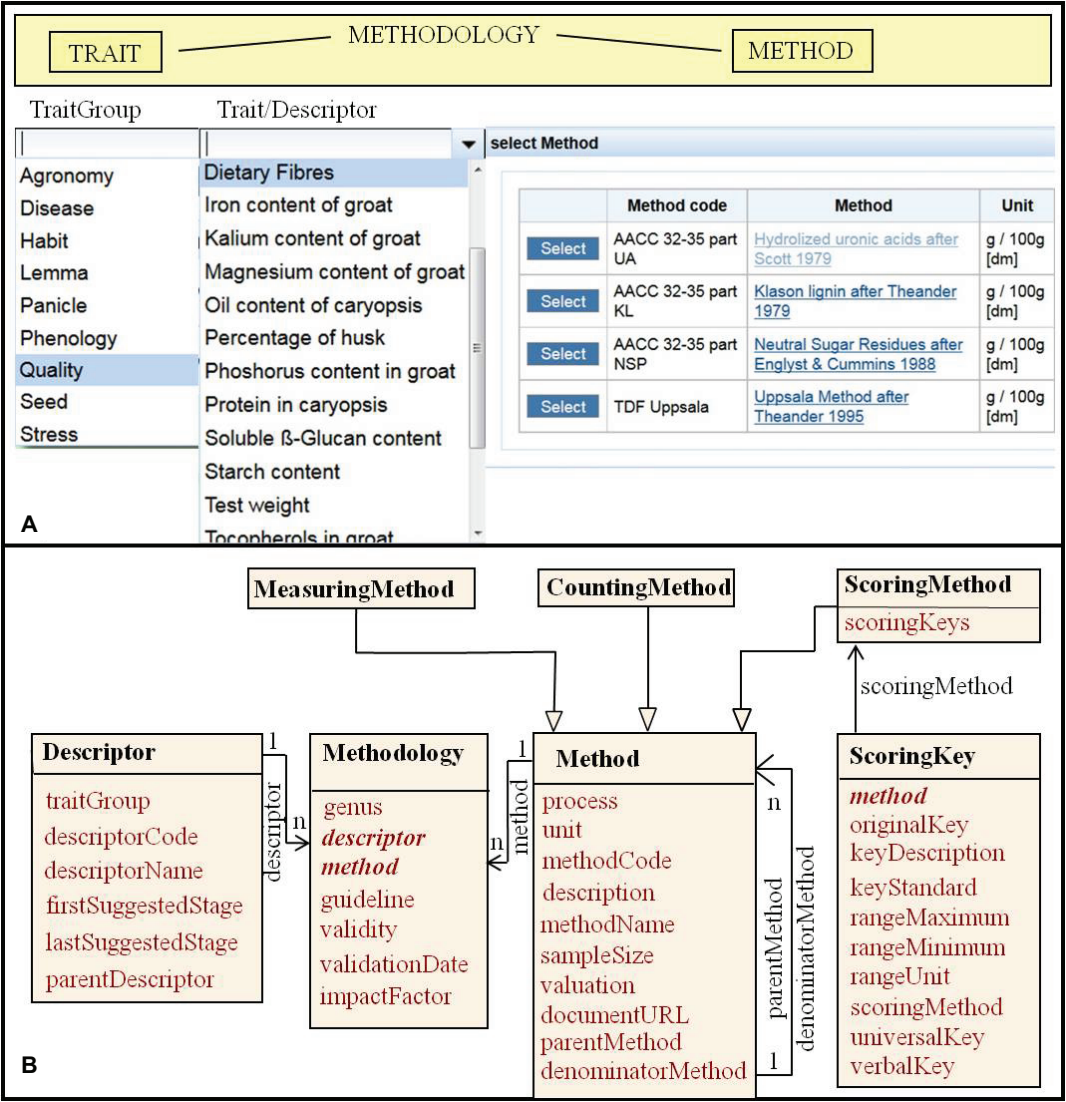
Trait methodology context data: **(A)** User interface, providing choice boxes for trait group or plant part, trait (as represented in trait descriptor lists), and analytical method for hierarchic search. **(B)** Entity/class model representing a trait descriptor and different types of observation methods including classification keys (scoring keys), composed to a methodology.

Main targets for breeding and crop research are phenotypic traits and their combinations (pyramidization) in a crop ideotype. Kattge et al. (2011) refer to a definition of traits as morphological, physiological, or phenological features measurable on individuals from cell to whole organism level. Descriptive context information for traits in plant breeding refers to protocols reflecting requirements in cultivar registration and evaluation of value for cultivation and use. Guidelines are available as descriptor lists by Bioversity International and preceding organizations (e.g., IBPGR, 1985; IPGRI, 1991; Alercia, 2013), by the International Union for the Protection of New Varieties of Plants (e.g., UPOV, 1994, 2008) and in trait ontologies now online for various crops (Shresta et al., 2010; Bioversity International, 2011; Arnaud et al., 2012). They provide also some suggested observation methods. For morphological characterization, their methodological information is mostly sufficient. For complex traits evaluating the value for cultivation and use (yield, resistance, and quality), various analytical methods are available and subject to methodological progress. Standard analytics for phytochemical traits are given, e.g., by the International Association for Cereal Science and Technology (2018). van Evert et al. (1999) in their data model dwell into details of equipments and their configuration. This could be useful for labs with similar machinery, but is not of general interest. It could be described in a method description or with links to (standard) methodology references. We compose trait descriptors and observation methods in a methodology class (Figure 3B). Krajewski et al. (2015) mention trait, method, and scale as foundational parts of an observation variable. To select, sort, or weight traits, we consider the phenological stages recommended to observing them, the type of observation (measurement, count, score), and units or coding schemes (scoring key) important. Specializations of a general method class represent different types of methods with different data type output (Figure 3B). Methods can be developed from each other (parent) or complement each other (denominator). We call the application of a methodology in an experiment (over all plots at a certain date) a measurement (Figure 2). Plant (species and cultivar) specific input parameters of crop models (Jones et al., 2003) should be considered in trait definitions for phenotypic information systems and in crop ontologies.

Easy search for combinations of trait expressions is crucial for a breeding information system. It is well supported by an observation table linked to trait metadata in a way enabling hierarchical search along an agreed categorization of traits and observation methodology, as given by trait ontologies (Bioversity International, 2011). These cover mostly three levels of search from a group of traits (abiotic and biotic stress tolerance, agronomy, morphology, phenology, physiology, quality) to defined single traits as outlined in descriptor lists (cf. UPOV, 2011; Alercia, 2013) and methodological details, e.g., units of measurement or definition of categorical scores (Figure 3A). This could also reflect the entity quality model of trait definition (Krajewski et al., 2015) with trait group relating to an entity (plant part) and trait to a quality.

### Context as Key to the Interpretation of Phenotyping Data

To improve the precision of statistical inferences for prediction and for a better understanding of genotype environment interactions, information on the context under which phenotypic data have been collected (experiment design, soil, biotic and abiotic interferences, treatments, and agronomic interventions) is required (Tinker and Yan, 2006; Ćwiek-Kupczyńska et al., 2016). It is needed to properly consider experimental factors and covariates in statistical analysis (Piepho et al., 1998; Kropff and Struik, 2002; Malosetti et al., 2013; Crossa et al., 2015). Crop research ontology (Bioversity International, 2011) and MIAPPE (Ćwiek-Kupczyńska et al., 2016) give a comprehensive overview. Kattge et al. (2011) stress the importance of covariates to understand the heterogeneity of trait expressions in an ecological context, to filter and to classify observation data. For a mechanistic understanding of crop performance, parameter requirements of crop models (Jones et al., 2003; White et al., 2013) are to be met. If context is overly complex, or requested attributes do not match the situation (e.g., growth chamber vs. field studies), it tends to be ignored or to be used improperly (Tinker and Yan, 2006).

**Figure 4:**
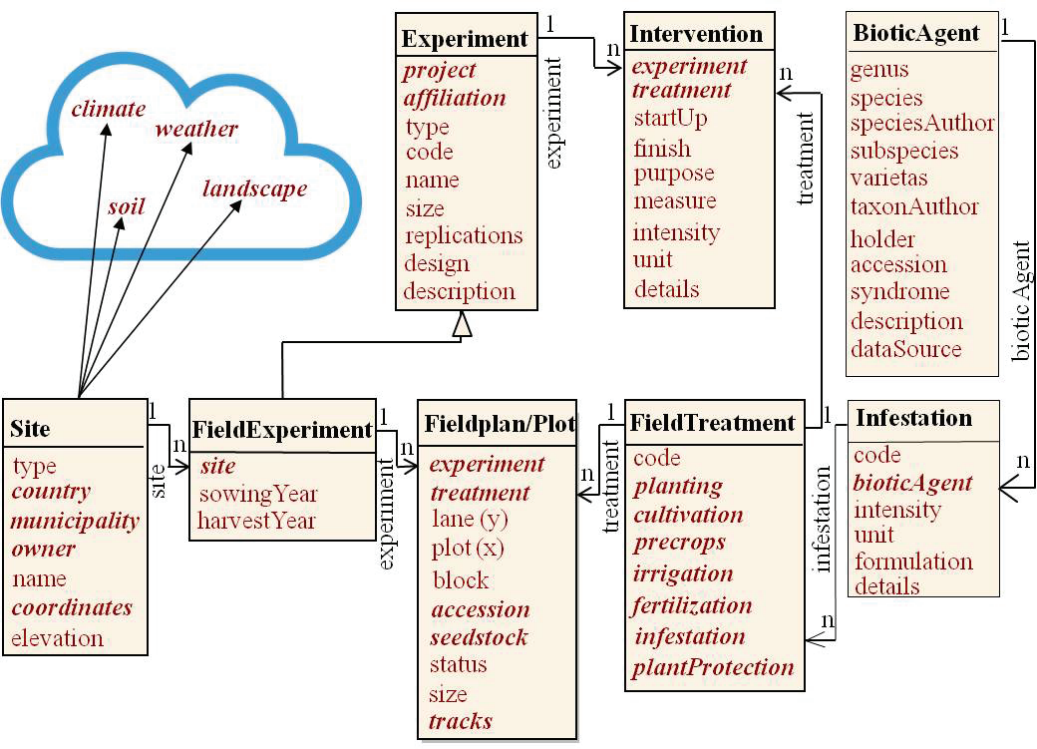
The field experiment context. Items in bold italics show foreign keys referring to other classes or modules. Important information might be available in the science cloud from other domains, e.g. geography (soil, site and landscape information) or meteorology.

Figure 4 depicts a data model for context information referring to measurements taken in a field plot or from samples thereof, covering the experiment data set in White et al. (2013). It treats a field experiment as specialization of a general experiment class. Further specializations could be greenhouse, growth chamber (Köhl et al., 2008; Fabre et al., 2011), laboratory, or other experiments. In MIAPPE (Ćwiek-Kupczyńska et al., 2016), a field or greenhouse study (comparable to experiment) extends a basic study. The Natural Diversity schema (Jung et al., 2011) models similar specializations as experiment types indicated by a respective attribute. The experiment super-class holds basic relationships to project, affiliations, and randomization design. We identify an experiment by project, institute, and specializing codes with the initiation date. Typical field randomization designs are block, latin square, lattice, or augmented designs. These are implemented through randomization in a field plan, which represents the block structure (Krajewski et al., 2015).

Plots are the experimental units (Tinker and Yan, 2006; Heckenberger et al., 2008) in a field experiment, arranged in a two-dimensional matrix (lane, plot) and populated with accessions and treatments. As van Evert et al. (1999) point out, plots can be structured on multiple levels by the design. Here the block attribute implements the first stratum below experiment and allows for all designs developed out of the block design, e.g., augmented block designs (Federer and Raghavarao, 1975). Higher level strata, e.g., for lattice designs could be implemented with further attributes in the field plan class. We consider one-site/one-season field experiments (called “test” in Tinker and Yan, 2006) and treat multi-location/multi-season field experiments as series or sets of field experiments held together within a project.

Distinguishing for a field experiment is the experiment site, identified by administrative (country, municipality) and geographic descriptors (geographic coordinates and elevation). Most of the environmental input variables required in DSSAT models (Jones et al., 2003) relate to the site. Data on landscape, soil, climate, and weather would be preferably linked from special geo-ecological information systems (symbolized by the cloud in Figure 4), e.g., as GIS layers by virtue of the geographic coordinates given. Field experiments undergo agronomic interventions (van Evert et al., 1999): pre-crops, soil-cultivation, sowing regime, fertilization, irrigation, and plant protection. These may be constant for the whole experiment or applied differently as experimental (variable) treatments (Krajewski et al., 2015). Fabre et al. (2011) have treatment attributes scattered in experiment, plot, and instruction tables. We collect them as attributes into a treatment (here field treatment) class. van Evert et al. (1999) represent treatments as combination of factor/level pairs, like Jung et al. (2011), in a vertical design. This gives maximum flexibility in treatment definition, but less guidance on important aspects of treatments to consider. Deficits in use of this model are mentioned by van Evert et al. (1999). We used it in the intervention table, but in addition relate pre-defined important aspects in the treatment class (planting, cultivation, pre-crop, irrigation, fertilization, infestation, and plant protection) to separately modeled details for these aspects. It is exemplified here with the infestation, which models artificial inoculation, e.g., with diseases, pests, or symbionts (biotic agents).

### Pictures as Increasingly Important Phenotyping Documents

Images are increasingly used for non-destructive, high-throughput phenotyping in plant research and breeding programs, with potential for observation in high temporal resolution (Walter et al., 2015; Araus et al., 2018). Images give multidimensional information (e.g., on shape and color) and allow decoupling of sampling and automatable analysis. Simple imaging tools are widespread available (Lobet et al., 2013). Image data management, image analysis, and result visualization are required (Klukas et al., 2014). Publicly available large and well-curated image datasets are imperative for the progress in image analyses (Fahlgren et al., 2015). Large-volume image data will remain a constant for most plant phenomics experiments and require image analysis tools on one hand and image storing and cataloguing on the other (Knecht et al., 2016).

**Figure 5:**
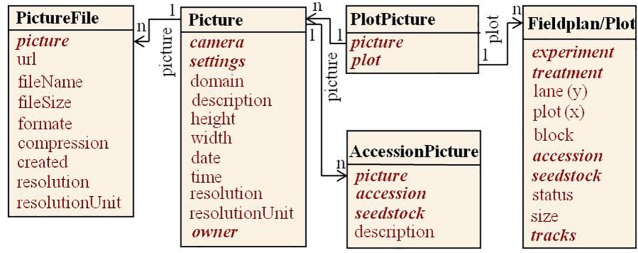
Pictures in our field experiment context relate to plots in a field plan and/or to PGR accessions. They are accessible through picture files, e.g. *via* URLs on web servers.

Specialized systems for image analysis have been described (Lobet et al., 2013; Klukas et al., 2014; Knecht et al., 2016; Gehan et al., 2017). Here we follow a concept to integrate images into the field experiment documentation linking image data by a uniform resource locator (URL) to plot level information of a field experiment (Figure 5). Image files can be stored on a file server or an open image repository and are related to specific plots in a field plan (n:m *via* table PlotPicture) and/or to specific accessions (n:m *via* table AccessionPicture). They are called by their uniform resource locator (URL) over the internet to be displayed with the web application (Figure 8). Metadata on camera and camera settings are automatically read into a picture table, file type, and attributes into a picture file table. Additional information, as a description, can be given by the user.

## Implementation Examples in European Central Crop Databases and Project Information and Management Systems As a Proof of Concept

Data and object models, as described above, have been developed for two ECPGR Central Crop Databases (CCDBs). The European *Avena* Database^2^ and the International Database for *Beta*^3^ list passport, characterization, and evaluation data for 34,541 and 10,613 genebank accessions, respectively (Table 1). Projects for characterization and evaluation of these resources have been initiated within the frame of regulations EC1467/97 (projects CT 95–42 and CT 99–196), and EC 870/2004 (project AVEQ). Currently, 163 descriptors (traits) from various descriptor lists (IBPGR/IPGRI/Bioversity, UPOV, COMECON) are compiled in the *Avena* database and 117 IPGRI descriptors in the *Beta* database (cf. Table 1 and Figure 3B for the data model). For 3,134 of the *Avena* accessions 169,799 phenotyping points (field experiment means) have been determined with 112 methodological approaches (specific methods for trait observation, cf. Figure 3) in the EADB, and for 1,750 *Beta* accessions 36,541 data points with 123 methodological approaches in the IDBB.

**Table 1:** Quantitative representation of important data types in the European Avena Database (EADB), the International Database for Beta (IDBB) and the AVEQ project database.

	Accessions	Holders	Descriptors (Traits)	Methods	Experiments	Observations	
						Raw	Aggregated
EADB	34,541	35	163	372	56	240,019	169,799
IDBB	10,613	29	117	141	123	–	36,541
AVEQ	667	39	-^1^	-^1^	25	257,148	

1 Used from EADB *via* database link.

Databases are implemented in the relational database management systems Oracle and MySQL. These can be directly accessed by statistical analysis software (SAS, R) *via* open database connectivity (ODBC) and respective structured query language (SQL) modules. Web applications to search the CCDBs have been developed in PHP. For a project on nutritional quality traits of oat genetic resources (AVEna genetic resources for Quality in human consumption, AVEQ, http://aveq.julius-kuehn.de), web applications for management and data acquisition in multi-site phenotyping of genetic resources, bridging the *Avena* CCDB and a project database have been developed in Java (JEE5/6, JSF, JBoss Seam) technologies and used to coordinate field designs and template based data acquisition at 11 European field experiment sites and to upload 257,148 evaluation data points (raw data) for 667 accessions in 33 traits with 75 observation and analytical methods, as have been generated in this project (Morcia et al., 2013; Murariu et al., 2013; Redaelli et al., 2013, 2016; Tumino et al., 2016). Workflows for online management and data acquisition have been developed in this project database.

## Implementation of Work Flows For Characterization and Evaluation of Plant Genetic Resources

Standard input and output by clearly defined workflows have been identified as preconditions for effective breeding with automated data processing. Lang et al. (2001) mention workflows to plan field experiments, which include selection of target lines and field plan generation, preparation of sowing lists, labels, and field books. Systematic form-based data collection effected by downloadable data gathering templates lead to more comprehensive and rigorous data recording (Tinker and Yan, 2006). We describe four work flows in experiment planning and documentation, which have been implemented, besides others, with the AVEQ web applications: field plan generation, field book and observation templates generation, upload of results from spreadsheets (e.g., MS Excel), as provided by mobile data acquisition tools or laboratory devices, and upload of photographs.

### Field Plan Generation

**Figure 6:**
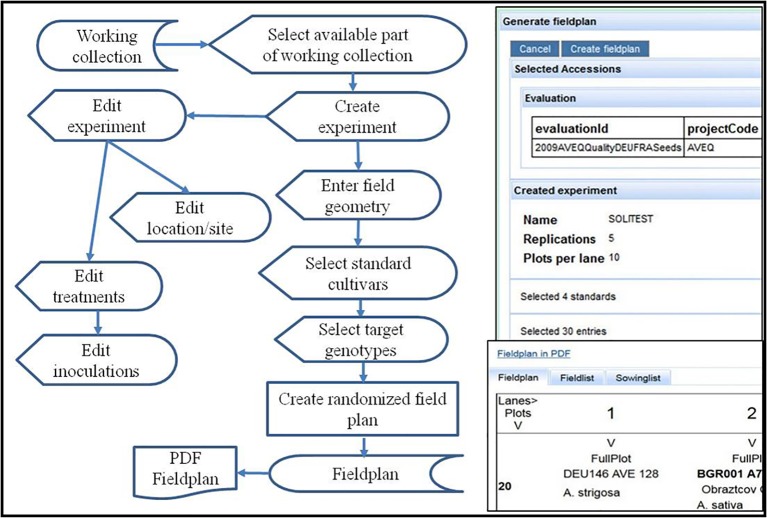
Flow diagram and screenshot of field plan generation. It starts with a working collection pre-defined in a project for a specific evaluation activity. The screen shot on the right shows the form to enter field design and geometry (limited by the possible number of plots per sowing lane) to define the field experiment after selection of standard and target cultivars (entries) for an augmented block design. Part of the field plan with two plots is shown in the lower part of the screenshot on the right.

This workflow (Figure 6) starts with a predefined and available (sufficiently multiplied) part of a project working collection to arrange it in a randomized field plan for a specific field experiment at a certain location in a certain year. The experiment will be created by entering basic attributes as the experiment name, the intended design, the number of replications (for the replicated part of the accessions in case of an augmented design), and the number of plots available for one sowing lane (depending on the field size). These parameters are necessary for the construction of a field plan. Later on experiment details (treatments, experiment site) can be edited or updated.

Sets of standard cultivars are essential for comparing results over experiments (Blake et al., 2012). They should be used over long periods of time and optimally represent defined trait expressions (susceptibility, resistance, high or low expression of a trait of interest). In screening experiments often only standard cultivars are grown in replications, forming the backbone for the statistical analysis, while the tested accessions are not replicated, e.g., in augmented designs (Federer and Raghavarao, 1975). The web application allows to select standard cultivars from a pre-defined list first, and then the selection of accessions from a working collection in the same manner. It also allows the integration of special plots for orientation or checking the correctness of the sowing operation. The field plan can be displayed as matrix or as list, both downloadable in PDF format.

### Generation of Field Books and Observation Templates

**Figure 7:**
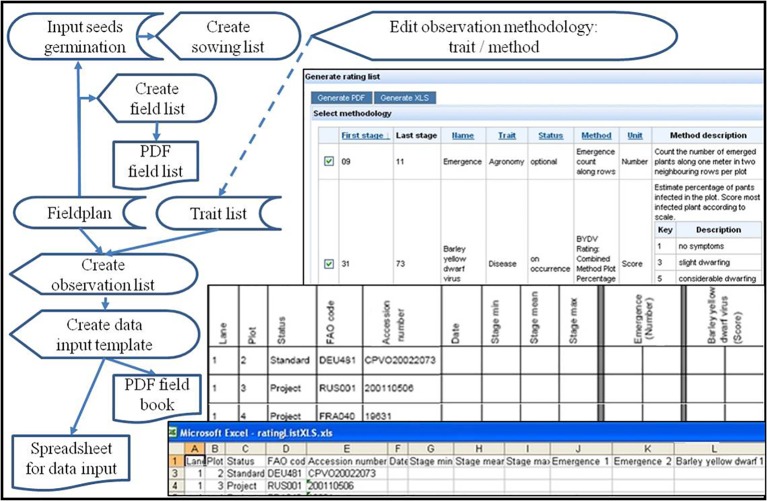
Flow diagram and screenshots (on the right) of field book generation: The upper screenshot (“Select methodology”) shows a list of trait observation methodologies ordered by recommended phenological stage to observe it, and allows to check them for including them as columns into a PDF (middle screenshot) or a spreadsheet template (lower screenshot). These integrate the selected trait observations with the field plan to give predefined lists for taking notes in the field manually or with mobile digital assistants.

Based on a field plan lists and templates for sowing and data acquisition can be generated in paper (PDF-file) or digital spreadsheet form. This comprises a group of work flows leading to sowing lists, field lists, lists, and templates for taking observations in the field (Figure 7). Sowing lists include the amounts of seeds needed based on germination test results. Field lists list all plots with accessions and treatments for demonstration purposes. Preparation of lists and templates for phenotyping data acquisition in the field starts with a check list of agreed traits to be observed (Screen “Select Methodology” in Figure 7). Target traits can be sorted according to phenological stages recommended for observation of the traits. So, they can be selected (checked) to fit the phenology encountered in the field. Lists for taking notes in PDF and spreadsheets for digital data input are created for download by combining a field list with the selected traits and respective methodology, which is explained by a methodology description on the first page of the PDF list.

### Map Spreadsheet Data for Import

**Figure 8:**
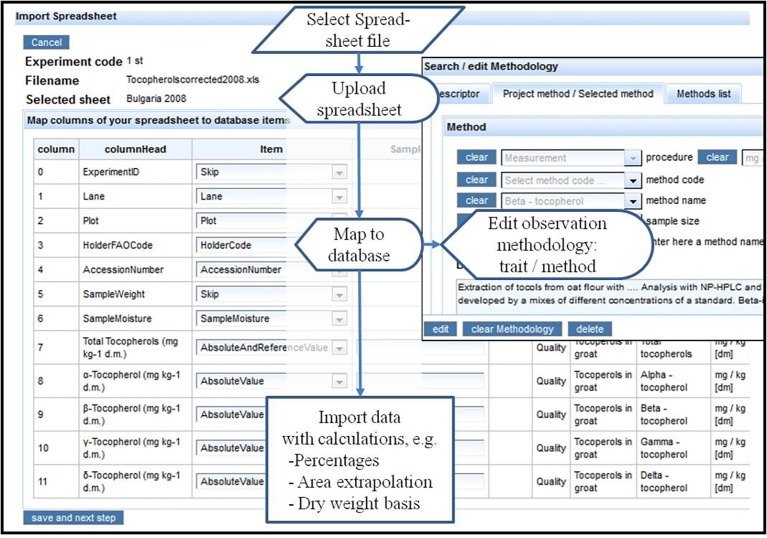
Flow diagram and screenshot of spreadsheet data import. The screenshot on the left shows spreadsheet column heads, which can be mapped to attributes in the database. If an observation item was entered (absolute value, percentage, score, etc.), the methodology form pops up (upper right screenshot) to prompt the user to select an observation methodology, which will be mapped to this spreadsheet column (see lower right part of the screenshot).

Tinker and Yan (2006) address populating a crop database with data from spreadsheets, which are normally produced with mobile data acquisition or other devices in fields and labs. They mention solutions with standardized templates (cf. previous workflow) or walking the user through the mapping of spreadsheet columns to database objects. The latter approach has the advantage that various formats and not only the standard template can be imported into the database. Yan and Tinker (2007) describe an implementation with MS Access. A screenshot and flow diagram for the implementation in the AVEQ web application is shown in Figure 8. A HTML table (Figure 8, left screenshot) shows columns and column heads of the uploaded spreadsheet and provides combo boxes to select predefined database objects (items = identifiers or trait expressions). The items (attributes) automatically relate to the appropriate tables. When an observation type (absolute value, percentage, score) is selected, the methodology form (Figure 8, right screenshot) pops up prompting the user to select or input trait and method (observation methodology) referred by the respective spreadsheet column. With this mapping protocol (which is stored in the database), the spreadsheet data are automatically imported into the observation table with appropriate links to the traits, to experiment (which has been entered and selected in advance) and the treatments (currently only genotypes). The application checks against importing the same spreadsheet twice and for consistency of genotypes in plots between field plan of the selected experiment and the uploaded data. It allows deleting the previous upload and replacing it by a corrected one.

### Upload Pictures

**Figure 9:**
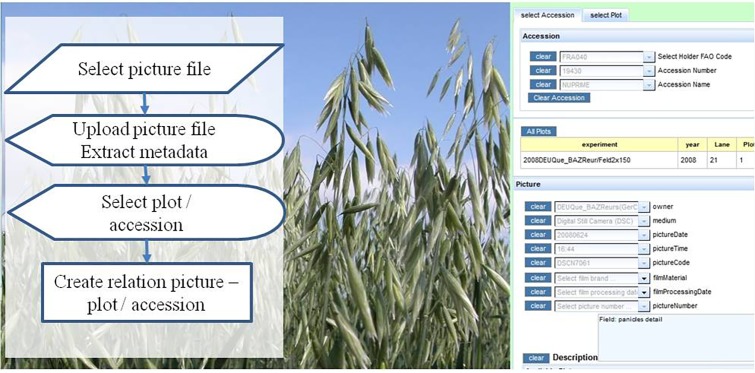
Flow diagram and screenshot of picture upload.

Image files can be linked into the web application (Figure 9). Metadata for these images, including relations to a specific accession or plot, are stored in the database. For uploading, a picture can be selected from the local file system. It will be copied into a directory of a web server, and metadata (including the URL for its display) are written into the database. The user is prompted to select a plot in a field experiment and/or an accession. This information will be displayed together with the picture. Pictures can be searched by taxonomy and experiment related information will be displayed along with the picture.

## Conclusion

This study develops, out of the requirements in statistical analyses and crop simulation modeling, highly comprehensive ER and class models for plant genetic resources characterization and evaluation (phenotypic) data acquisition in field experiments. They have served for more than 10 years in the framework of ECPGR Central Crop Databases and in PGRFA characterization and evaluation project information and management systems. They can serve as blueprints for future developments with current software technologies in similar contexts, especially within EURISCO, which is currently superseding the Central Crop Databases, and within the on-farm PGRFA communities.

Modeling at the different levels of abstraction from the database to the statistical model (Figure 1) is considered, using object oriented software development approaches and tools, confirming Papajorgji et al. (2004), that these can be useful not only in simulation modeling, but generally in conceptualization of scientific knowledge. Databases and information systems are suggested as integral tools of scientific work, and software modeling tools, like ER and class diagrams as useful for structuring scientific results. Additionally several objects, attributes and features are shown, which are not yet available in any of the other systems currently available for the documentation of phenotyping data.

Over-generalization and over-normalization often impede modularization along a specialization of expertise and thus the reuse of software components in neighboring domains. These are evident, if entities and classes do not correspond with objects and concepts well known in the domain. To our knowledge, there is currently no attempt in the literature on biological databases, showing such clearly the possible congruence of domain concepts, database entities, and software model classes at least in the persistence and business tiers of an information system. It should encourage scientists to take more influence in scientific software development and thereby enhancing usability and reuse of scientific databases and software. The entity relationship, class, and flow models shown in the figures can be useful as a blue-print for developing phenotypic information systems in crop science.

## Author Contributions

CG basically designed the database and web applications, reviewed the respective literature, and wrote the manuscript. SU refined models and design and did a great part of software implementation. Both authors read and approved the final manuscript.

### Conflict of Interest Statement

The authors declare that the research was conducted in the absence of any commercial or financial relationships that could be construed as a potential conflict of interest.

## Footnotes

^1^http://www.obofoundry.org

^2^http://eadb.julius-kuehn.de

^3^http://idbb.julius-kuehn.de
